# EEG in Neurorehabilitation: A Bibliometric Analysis and Content Review

**DOI:** 10.3390/neurolint14040084

**Published:** 2022-12-16

**Authors:** Athanasia Tsiamalou, Efthimios Dardiotis, Konstantinos Paterakis, George Fotakopoulos, Ioannis Liampas, Markos Sgantzos, Vasileios Siokas, Alexandros G. Brotis

**Affiliations:** 1 Faculty of Medicine, School of Health Sciences, University of Thessaly, 41334 Larissa, Greece; 2 Department of Neurology, General University Hospital of Larissa, 41110 Larissa, Greece; 3 Department of Neurosurgery, General University Hospital of Larissa, 41110 Larissa, Greece

**Keywords:** EEG, stroke, traumatic brain injury, neurorehabilitation, brain–machine interface

## Abstract

Background: There is increasing interest in the role of EEG in neurorehabilitation. We primarily aimed to identify the knowledge base through highly influential studies. Our secondary aims were to imprint the relevant thematic hotspots, research trends, and social networks within the scientific community. Methods: We performed an electronic search in Scopus, looking for studies reporting on rehabilitation in patients with neurological disabilities. We used the most influential papers to outline the knowledge base and carried out a word co-occurrence analysis to identify the research hotspots. We also used depicted collaboration networks between universities, authors, and countries after analyzing the cocitations. The results were presented in summary tables, plots, and maps. Finally, a content review based on the top-20 most cited articles completed our study. Results: Our current bibliometric study was based on 874 records from 420 sources. There was vivid research interest in EEG use for neurorehabilitation, with an annual growth rate as high as 14.3%. The most influential paper was the study titled “Brain-computer interfaces, a review” by L.F. Nicolas-Alfonso and J. Gomez-Gill, with 997 citations, followed by “Brain-computer interfaces in neurological rehabilitation” by J. Daly and J.R. Wolpaw (708 citations). The US, Italy, and Germany were among the most productive countries. The research hotspots shifted with time from the use of functional magnetic imaging to EEG-based brain–machine interface, motor imagery, and deep learning. Conclusions: EEG constitutes the most significant input in brain–computer interfaces (BCIs) and can be successfully used in the neurorehabilitation of patients with stroke symptoms, amyotrophic lateral sclerosis, and traumatic brain and spinal injuries. EEG-based BCI facilitates the training, communication, and control of wheelchair and exoskeletons. However, research is limited to specific scientific groups from developed countries. Evidence is expected to change with the broader availability of BCI and improvement in EEG-filtering algorithms.

## 1. Introduction

After severe traumatic brain injury (sTBI) and spinal cord injury (SCI), stroke, and other neurodegenerative disorders, patients frequently experience significant neurological disabilities. Traditional rehabilitation focuses on teaching compensatory skills and allows the patient to return home as soon as possible but does not seem to reduce impairment [1,2]. Alternatively, functional recovery might result in more-sustainable outcomes, as it has been associated with a long-term reduction in impairment and offers an improvement in quality of life [1,2]. Thus, neurorehabilitation has recently shifted toward more-active paradigms, particularly in patients with motor and communication disabilities [1,2]. Several approaches can improve motor learning, including massed and task-specific practice, multisensory stimulation, and motor imagery [1,2]. Similarly, intensive speech and language therapies, including constraint-induced aphasia therapy, which activates both the linguistic and the concordant motor circuits, can rapidly improve language performance [1,2].

Lately, brain–computer interfaces (BCIs), communication systems that recognize users’ commands only from the brain signals and react according to them, have been used in inpatient rehabilitation, with promising results [3,4]. Various invasive and noninvasive modalities, such as electroencephalography (EEG), near-infrared spectroscopy (NIRS), and electrocorticography (ECoG) [3,4], are frequently used to identify proper brain signals. High-density EEG constitutes an advanced and quantitative technique based on the multichannel recording of the brain’s electrical activity to localize underlying brain structures [5]. To the best of our knowledge, there is a paucity in the pertinent literature on quantitative studies reviewing the role of EEG in neurologic rehabilitation.

In our current study, we used bibliometrics, a systematic and reproducible way to review the literature on the basis of the statistical analysis of highly cited records, to analyze and evaluate the literature on EEG in neurorehabilitation [6,7]. Bibliometrics are based on the assumption that the number of citations could reflect the impact or value of a particular article to a certain extent. We primarily aimed to identify the knowledge base through highly influential studies. Our secondary aims were to examine the relevant research front, focusing on active authors, thematic hotspots, and research trends. We also aimed to track the pertinent social networks within the scientific community regarding co-operations between institutions, countries, and authors. By referring to this article, readers can learn literature trends and characteristics of scientific documents to gain insights to guide future studies.

## 2. Material and Methods

We conducted a bibliometric analysis according to the workflow recommended for science mapping (M. Aria and C. Cuccurullo), using the statistical environment R, the biblioshiny interphase (package bibliometrix), and VOSviewer [6,7]. Bibliometrix is a popular tool used for bibliometric analysis, particularly in health sciences [6]. Since we gathered literature data without involving any patients, the current study was exempted from Institutional Review Board (IRB) approval and patient informed consent [8].

### 2.1. Search Strategy

Our electronic search was carried out in the medical database Scopus. We preferred the particular database because it is a broad database with many records and permits the extraction of scientometric metadata [9,10]. To avoid duplicates and because of inherent software limitations, we limited our search to a single database. In this study, *rehabilitation* was defined as the field of science involved in neurologic recovery after sTBI, SCI, stroke, and other central nervous system disorders, such as amyotrophic lateral sclerosis (ALS) and locked-in syndrome (LiS) [11]. Neurologic rehabilitation included the neural repair, regeneration, and dynamic reorganization of functional neural systems, manifested by increased awareness and by return to function and freedom [11].

### 2.2. Eligibility

We aimed to search titles, abstracts, and keywords for “rehabilitation”, “neurological disorders”, and “electroencephalography” in any form and combination (Table 1). The search was limited to studies written in English in any form and without further limitations on the publication date. We intended to gather studies with significant impact, and therefore, we decided to include any publication type, such as reviews, editorials, letters to the editor, and conference abstracts [12]. Our limitation in the manuscript language was not expected to change our results, because English written articles have the largest penetration in health sciences [13]. We included all records resulting from the electronic search.

### 2.3. Data Collection

All citation data, bibliographical information, abstracts, and keywords of the eligible records were downloaded using the BibTeX format. We retrieved the article title, names and number of authors, year and journal of publication, Scopus citation count, and the corresponding author’s country for our bibliometric analysis. Data were loaded on *biblioshiny* and analyzed without any further filtering [6].

### 2.4. Data Analysis

The current study’s data analysis occurred in two steps, using descriptive analysis and a network extraction process. We performed a descriptive analysis using standard competition ranking to retrieve evidence on the most productive authors and countries, the most cited papers, the most frequent journals, and the most common author’s keywords [6]. In the network extraction process, we performed three subanalyses, including a collaboration analysis according to universities and countries, a cocitation analysis based on authors, and a word co-occurrence analysis according to the author’s keywords [6]. To assess the extent of international collaborations, we used the indices of single country publications (SCP), multiple-country publications (MCPs), and the ratio of SCP to MCP [6,14]. In SCP, all authors belonged to the same country, representing intracountry collaboration [14]. On the contrary, authors belonged to different countries in MCP, and such publications represented an international collaboration [6,14].

### 2.5. Data Synthesis and Quality Assessment

The results were presented in tables. Trends and temporal data were visualized in burst detection and simple time series plots. Word proximity maps were used to present the word co-occurrence analysis, the university collaboration analysis, and the author cocitation analysis. Geospatial data and conceptual structures were shown in geographic maps and cluster strings. Finally, after gathering and reading the full text of the top-20 most cited studies, we performed a narrative literature review.

## 3. Results

### 3.1. Literature Search

**Figure 1:**
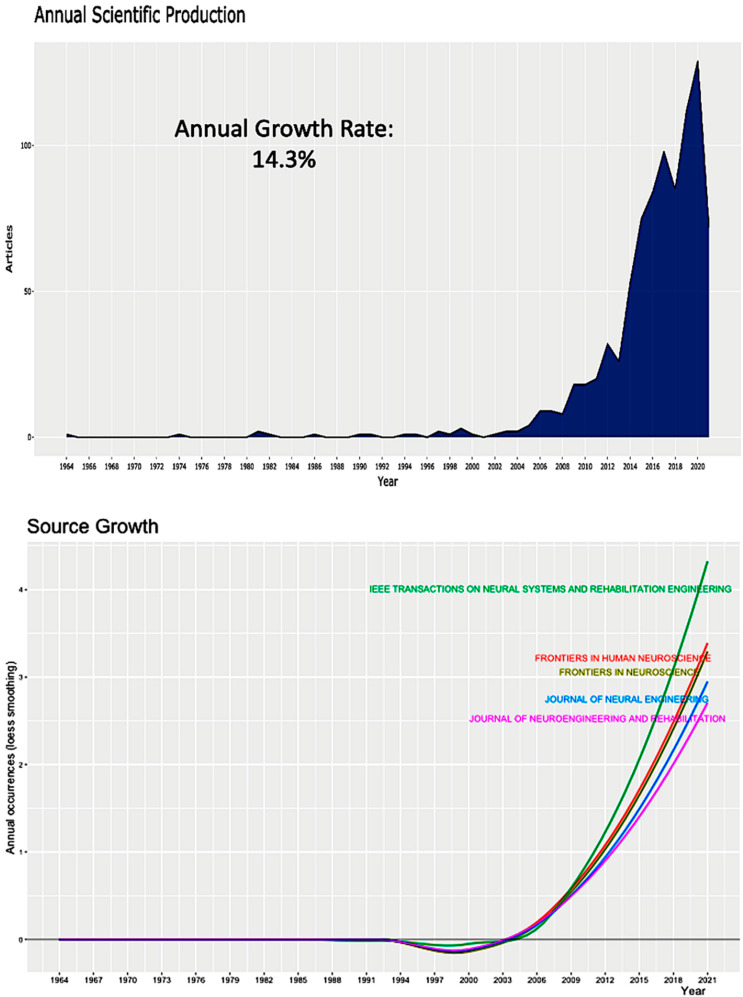
According to the annual scientific production (top) and source growth (bottom), there is rising scientific interest in using EEG in neurorehabilitation.

The electronic search in Scopus resulted in 874 articles from 420 sources, including journals, special issues, and books (Table 1). Among the gathered documents, there were 456 original studies, 145 conference papers, and 119 reviews. With a total of 41,104 references, there was an average of 21.63 citations per document, and the average years after publication were as high as five. The numbers of Scopus and authors’ keywords were 6146 and 1946, respectively. We recorded 3589 authors, with 0.24 documents per author and 5.29 coauthors per document. There was rising interest in EEG use in neurorehabilitation, with an annual growth rate as high as 14.3% during the past 10 years (Figure 1, top).

### 3.2. Top-20 Most-Cited Documents

The list of the top-20 most cited articles is depicted in Table 2 [3,4,15,16,17,18,19,20,21,22,23,24,25,26,27,28,29,30,31,32]. The most cited document was “Brain-computer interfaces, a review” by Nicolas-Alfonso et al., (997 citations), followed by “Brain-computer interfaces in neurological rehabilitation” by J. Daly and J.R. Wolpaw (708 citations) [3,4]. In this list, 13 (65%) documents were reviews, six (30%) were research articles, and the remaining one (5%) was a symposium summary. Five studies (25%) involved stroke, whereas one article (5%) was related to sTBI, SCI, and ALS/LiS. Brain–computer interface (BCI) was the main topic in 11 (55%) studies. Finally, neurorehabilitation was used to improve motor function in 15 (75%) studies, whereas, in three (15%) studies and one (5%) study, neurorehabilitation was implemented to control machines and facilitate communication.

### 3.3. Top-20 Most-Productive Authors

**Figure 2:**
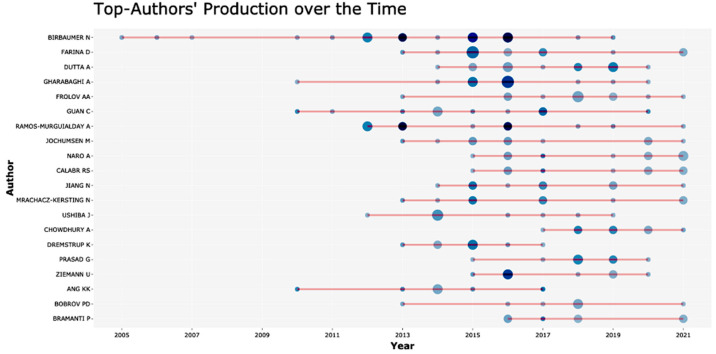
The figure shows the scientific production of the most influential authors.

The top-20 most productive authors are depicted in Figure 2, while most-cited countries, based on the first author’s affiliation, are shown in Table 3. G. Pfurtscheller, N. Birbaumer, and J.R. Wolpaw occupy the top three of the most cited authors, with 975, 875, and 501 citations, respectively.

### 3.4. Top-20 Journals

The gathered records were reported in 420 sources, and the top-20 journals were listed in Table 4. We witnessed an active source growth, particularly for the journals of *IEEE Transactions on Neural Systems and Rehabilitation*, *Frontiers in Human Neuroscience*, *Frontiers in Neuroscience*, and *Journal of Neural Engineering* (Figure 1, bottom).

### 3.5. Top-20 Most-Common Author’s Keywords and Word Trends

**Figure 3:**
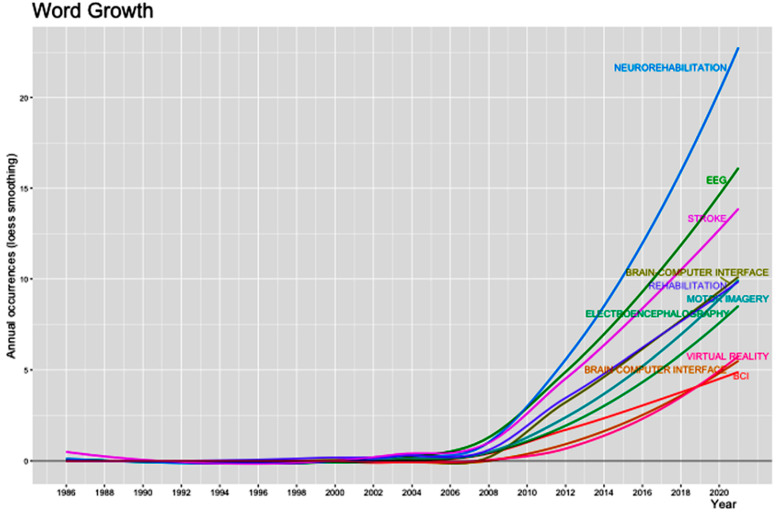
We noted important changes in the scientific trends with time, as recorded from the words’ growth (top).

The top-20 most common author’s keywords and the trends regarding word growth are depicted in Table 4 and Figure 3. Except for the words “rehabilitation” and “electroencephalography” and their derivatives, the term “brain–computer interface” predominated among the author’s keywords, with 180 occurrences in various forms. We also recorded a rising trend in all keywords since 2005. However, the trends changed over time, from the use of “function 9rganization magnetic imaging” to “brain–machine interface”, “motor imagery”, and “deep learning”.

### 3.6. Collaboration Analysis

**Figure 4:**
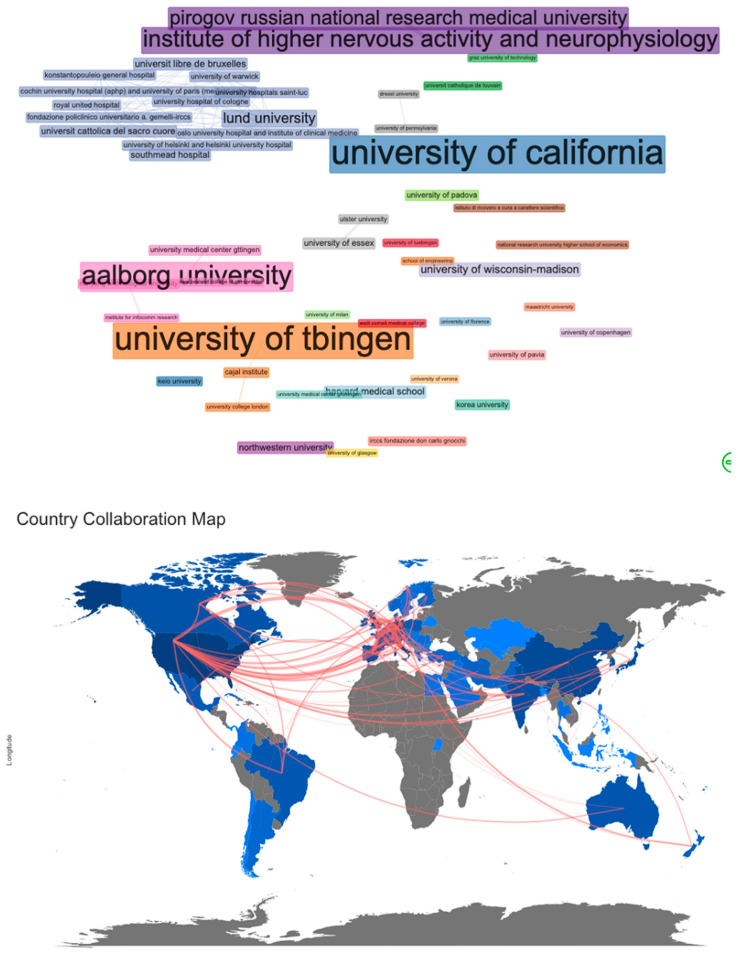
Significant international collaborations among institutions and countries on the use of EEG in neurorehabilitation.

According to the primary author, the US, Italy, and Germany were the topmost productive countries. According to the MCP/SCP ratio, many of the leading countries, such as the US (21%), Italy (19.78%), and China (16%), were limited in local co-operations. In contrast, countries such as Denmark (75%), Singapore (62%), Spain (61%), France (53%), Canada (47%), Australia (45%), Belgium (44%), and Germany (42%) were involved in more-extensive collaborations. Accordingly, the collaboration analysis map among institutions revealed a major co-operation network centered on the University of Lund (Figure 4, top). The remaining collaborations were limited to no more than a couple of institutions in every case.

### 3.7. Coword Analyses

On the basis of on the author’s keywords, the coword analysis identified four major clusters (supplement Table 5). The keyword “motor imagery” characterized the first and fourth clusters. “Virtual reality” and the “disorders of consciousness” prevailed in the second and third clusters.

## 4. Review Based on the Top-20 Most Cited Articles

The term “brain–computer interface” (BCI) refers to a hardware and software system that has been designed to control external computers or devices using cerebral signals [3,4,18,25]. In neurorehabilitation, BCI was used to assist severely disabled patients with sTBI, SCI, stroke, and ALS/LiS to interact with the environment [3,4,18,25,26], and it works in five stages: (1) signal acquisition, (2) preprocessing or signal enhancement, (3) feature extraction, (4) classification, and the (5) control interface [3].

In signal acquisition, several brain signals, either electrophysiological or hemodynamic, are invasively gathered or noninvasively detected before further amplification, filtering, and decoding using online classification algorithms [3,4,18]. The slow cortical potentials (SCP), sensorimotor rhythms, P300 event-related potentials, steady-state visual evoked potentials, and cerebral oxygenation levels are frequently registered for this purpose [3,4,18]. Noninvasive modalities such as EEG, functional magnetic resonance (fMRI), and NIRS were the most extensively studied tools in recording brain activity because their invasive counterparts (ECoG and intracortical neuron recording) have been associated with significant health risks, including microelectrode rejection, infection, and tissue damage [3,16,18,29]. The primary motor and the prefrontal cortices were the preferred brain targets for EEC-based BCIs and could be used in conjunction with NIRS in a hybrid technology [16,29]. In an experimental study, Lew et al. focused on the noninvasively recorded readiness potential, a SCP detected over the central medial areas [27]. The authors documented a high SCPs detection rate of 500 ms before movement onset. The absence of SCPs during the nonmovement intention period allowed the authors to conclude that it could be a valuable tool in neurorehabilitation [27].

Signal preprocessing or enhancement involves signal amplification and noise removal [3,4,25]. EEG signals generated by motor task imagery could be translated into external actions [22]. However, the processing of EEG signals, which directly affects classification accuracy, still represents a crucial challenge. They are susceptible to several factors, including the physical state, mood, posture, and external noise [22]. Kevric et al. compared three EEG signal processing techniques, the ﻿empirical mode decomposition, discrete wavelet transform, and wavelet packet decomposition, to decompose EEG signals in a BCI system and task classification [22]. The authors reported that the highest classification (92.8%) was achieved by combining multiscale principal component analysis denoising and higher-order statistics features extracted from wavelet packet decomposition sub-bands. The latter could be used to control external devices, including a wheelchair [22].

On the other hand, brain activity signals come in specific patterns, which need to be recognized, selected, extracted, and matched to the patient’s intention, using classification or regression algorithms [3,4,25]. Regression algorithms employ EEG features as independent variables to predict user intentions [3]. In contrast, classification algorithms use the features extracted as independent variables to define boundaries between the different targets in feature space [3].

The ultimate goal is to use EEG-based BCI and help patients with paralysis disabilities to communicate and control their environment, including external robotic devices and prosthetics, as in patients with ALS/LiS [3,4,17,18,26]. In addition, the recovery of neural function and motor function restoration in patients after stroke or SCI could be facilitated on the basis of rehabilitative BCIs in conjunction with virtual reality–assisted training and behavioral physiotherapy by inducing neural plasticity [3,4,18,20,23,26,28]. Indeed, the addition of BCI training to behaviorally oriented physiotherapy could induce functional improvements in motor function in patients with chronic stroke symptoms, without residual finger movements, and may open a new door in stroke neurorehabilitation [15]. Motor imagery represents a challenging method in rehabilitating patients with stroke symptoms by promoting the recruitment of the motor system for functional recovery [30]. It involves attempts to execute imagined movements using the plegic hand [30]. Ang et al. tried to ﻿investigate the ability of 54 patients who had a hemiparetic stroke to operate an EEG-based motor imagery BCI [30]. In addition, they compared EEG-based MI-BCI in conjunction with robotic feedback neurorehabilitation to robotic rehabilitation that delivers movement therapy in terms of motor improvement on the stroke-affected upper limb [30]. The authors reported significant gains in the functional scores in postrehabilitation and the two-month follow-up [30]. Of note, there were no significant differences between groups [30]. ﻿Motor outcomes could be potentiated using proprioceptive BCI in patients with residual proprioception and ﻿sessions of music-supported therapy [31,32]. However, there were cases where neuroplastic alterations were driven into maladaptive domains with significant adverse symptoms, such as phantom limb pain [28].

Equally important, EEG-controlled exoskeletons were designed as assistive devices for individuals with disabilities but could also be used in rehabilitation to assist or resist passive and active movement [17,26]. Assistive technologies provided support and balance during walking and whole-body navigation using a wheelchair [17,25,26]. Therapeutic devices target the improvement of physiological health by increasing physical activity and weight-bearing capabilities [17,26]. According to evidence that active contributions to the movement could be critical for encoding motor memory, Wagner et al. proposed brain-monitoring techniques during gait training to encourage active participation [23]. Thus, the authors compared the spectral patterns in the EEG during active walking and passive robot-assisted walking [23]. Independent EEG components were clustered across participants on the basis of their anatomical position and frequency spectra [23]. The authors provided evidence for significant cortical activation differences between active and passive robot-assisted gaits [23]. They noted the significant suppression of the mu, beta, and gamma rhythms during active walking, particularly compared with passive walking [23]. ﻿These differences depended on the phase of the gait cycle [23]. Similar differences were recorded in the right-hand area [23].

It seems that the use of EEG and BCI in neurorehabilitation was associated with significant limitations. Initially, there was a substantial lack in the literature of large randomized controlled clinical trials using invasive and noninvasive BCIs with long-term follow-ups in patients rather than healthy populations [3,18]. In addition, BCI systems must become safer, more reliable, cosmetically acceptable, user-friendly, and highly accurate [24]. The high cost of BCI technologies may raise ethical concerns, particularly in patients with ALS/LiS on ventilatory support [24].

## 5. Discussion

### 5.1. Overview of Our Findings

Our current manuscript presents a detailed analysis of the top cited articles on the use of EEG in neurorehabilitation. It can help clinicians and researchers understand the existing knowledge base, comprehend the current research front, and become acquainted with the underlying social/scientific networks. [*]We specifically identified the articles that served as landmarks in the field and the most influential authors. We preferred the number of citations as a criterion in ranking our articles among several other, including the date of publication and the h-index, because we believe that it is the most appropriate indicator of the scientific impact of an article in the field. Thus, we reviewed the pertinent literature on the basis of the most influential contributions. Likewise, we showed that the available research front originates from a limited number of institutions with an even smaller number of co-operations among them. Finally, it became evident that most research originates from affluent countries from Europe and from the US, with little to minimal contribution from Asia and the South America. BCIs constitute the connecting link between EEG and rehabilitation. This study seems to be the first study focusing on a specific topic. Therefore, there are no relevant studies that can be used for further comparison. Nevertheless, a series of thoughts have been elicited by the current findings and are presented thereunder.

### 5.2. Bibliometrics

Bibliometrics is a viable means to qualitatively and quantitatively review the literature [6,7,14,33]. It is an analysis of “big data” originating from literature databases, using artificial intelligence (AI) [34]. It is based on the concept that highly cited articles have a significant impact on the development of science [35]. Accordingly, we could identify the research background of the most active authors, countries and institutions, and journals [36]. The research trends and hotspots could be identified on the basis of the most frequent author’s keywords and their changes over time. Finally, various networks between authors, countries, and institutions could be recognized by using a word co-occurrence analysis.

### 5.3. Temporal Trends

Neurorehabilitation and control of external devices using EEG-based BCI were characterized by a recent rise of scientific interest and a high annual scientific production. Even though motor and communication disabilities have been around for as long as humanity has existed, this rise occurred in the past decade. Several scientific advances preceded, including the development of AI and several signal-filtering algorithms [37,38]. Thus, we recorded an exponential rise in scientific production since the early 2010s. There are probably two additional reasons why EEG in neurorehabilitation showed this late bloom. There was a shift in the rehabilitation approach from teaching patients to cope with disability toward improving the functional outcome by neurorehabilitation [3,4,25]. Indeed, over the past decades, rehabilitation intended to teach the patients tips and tricks on how to eat, dress, and independently move about. Nowadays, rehabilitation aims to improve the function of the paralyzed extremity by activating dormant or hibernating cerebral circuits to assist in walking and the execution of the activities of daily living. Furthermore, the use of EEG in neurorehabilitation demands a deep understanding of brain function and thorough computer science knowledge [39]. It seems that these disciplines only recently reached fruitful crossroads.

### 5.4. Journal Preferences

An analysis of the sources showed that studies on the use of EEG in neurorehabilitation are published in journals focusing on engineering and biomedical signals, rehabilitation, and neurosciences. These journals pioneer in hosting articles at the crossroads of neurosciences with other disciplines, particularly bioengineering. Indeed, the exponential rise in BCI research and the use of exoskeletons shifted authors and editors to journals merging neurosciences and engineering. Classical medicine and neurology journals are absent from the top-20 most cited journals.

### 5.5. Geographical Distribution

The current study demonstrated two significant controversies. To start with, the majority of the studies come from the affluent countries of the US and Europe. Asia and South America are represented to a lesser extent, with a minimal contribution from Africa. This map follows the rehabilitation requirements after stroke in affluent countries with older populations and less by craniospinal trauma in developing countries [40,41]. It remains to be shown whether a similar distribution exists in lower-income subgroups within the affluent countries. The second controversy is that the leading countries prefer a more self-sufficient approach instead of participating in extensive international collaborations. Indeed, it seems that “elite players” such as the US, Italy, and China prefer lonely paths. At the same time, smaller partners, such as Denmark, Singapore, Spain, and others, are more interested in international collaborations.

### 5.6. Document Type

In the present review, we focused on studies with the highest impact in the field. We decided to include all document types, in addition to original studies, as we noted that reviews and conference papers were among the highly ranked documents in our initial pilot searches. The presence of reviews in our study highlights the need to spare time and resources from exhaustive searches in a rapidly evolving field. In addition, the large number of conference papers among the highly cited documents shows that a significant part of the evidence has not been published in peer-reviewed journals, raising several questions on the reproducibility of the findings and the role of funding in research.

### 5.7. Limitations

Significant limitations characterized the current study. First, it was based on bibliographic data from Scopus because of the inherent limitations of the adopted software and to avoid duplicates, as explained earlier. As a matter of fact, the utilized software, Bibliometrix, is capable of analyzing the metadata from a single database. However, we selected Scopus because it is the single largest extractable medical database with the most useful scientometric data. Second, a bibliometric analysis lacks an in-depth analysis of the gathered articles. For this reason, we added a content review of the 20 most cited articles. Third, a higher citation index is not necessarily synonymous with better methodological quality and improved reporting clarity. In other words, a bibliometric analysis could include low-quality studies, but with a significant impact in the field. Likewise, our study could omit high-quality studies that are still immature to reach a high citation number. Therefore, the reader is cautioned to note that our results are expected to change, and regular literature updates are mandatory in the future. Finally, a bibliometric analysis may result in a limited number of irrelevant articles after reading the document’s full text. One (5%) irrelevant study was found among the top-20 results in our sample [19].

## 6. Conclusions

EEG constitutes the most significant input in brain–computer interfaces (BCIs) and can be successfully used in the neurorehabilitation of patients with stroke, ALS/LiS, sTBI, and SCI. EEG-based BCI facilitates training, communication, and control of wheelchairs and exoskeletons. However, research is limited to specific scientific groups from developed countries. In addition, there seems to be unpublished evidence with significant impact. Evidence is expected to change with the broader availability of BCI and improvement in EEG-filtering algorithms.

## Figures and Tables

**Table 1:** Main bibliometric characteristics of documents retrieved from Scopus.

Description	Results
Main Information about Data	
Timespan	1964:2021
Sources (journals, books, etc.)	420
Documents	874
Average years from publication	5.03
Average citations per documents	21.63
Average citations per year per doc	3.13
References	41104
Document Types	
article	546
book	1
book chapter	17
conference paper	145
conference review	4
editorial	18
erratum	1
letter	11
note	5
retracted	1
review	119
short survey	6
Document Contents	
Keywords Plus	6146
Author’s Keywords	1946
AUTHORS	
Authors	3589
Author appearances	4623
Authors of single-authored documents	40
Authors of multi-authored documents	3549
Authors Collaboration	
Single-authored documents	45
Documents per author	0.244
Authors per document	4.11
Coauthors per documents	5.29
Collaboration index	4.28

Search in Scopus: (TITLE-ABS-KEY (neurorehabilitation) OR TITLE-ABS KEY (rehabilitation AND neurological AND disorders OR TITLE-ABS-KEY (neurorehabilitation)) AND (TITLE-ABS-KEY (EEG) OR TITLE-ABS KEY (electroencephalography) OR TITLE-ABS KEY (electroencephalogram) AND (LIMIT TO (LANGUAGE, “English”)).

**Table 2:** Top-20 globally most-cited documents.

Paper	Year	Journal	Total Citations	Study Design	Clinical Entity	Main Topic	Use
Nicolas-Alfonso L and Gomez-Gill J [3]	2012	Sensors	997	Review	Multiple	BCI	Rehabilitation
Daly J and Wolpaw J [4]	2008	Lancet Neurol	708	Review	Multiple	BCI	Rehabilitation
Ramos-Murguialday A et al. [15]	2013	Ann Neurol	521	Research	Multiple	BCI	Motion
Naseer N and Hong K [16]	2015	Front Human Neurosci	483	Review	Multiple	BCI	Motion
Young A and Ferris D [17]	2017	IEEE Trans Neural Syst Rehabil Eng	305	Review	Multiple	Exoskeleton	Motion
Chaudhary U et al. [18]	2016	Nat Rev Neurol	293	Review	Multiple	BCI	Communication
Kos D et al. [19]	2008	Neurorehabil Neural Repair	274	Review	MS	MS	Rehabilitation
Rizzolatti G et al. [20]	2009	Nat. Clin. Pact. Neurol	268	Review	Multiple	Mirror neurons	Rehabilitation
Donati A et al. [21]	2016	Sci Rep	197	Research	SCI	BCI	Rehabilitation
Kevric J and Subasi A [22]	2017	Biomed Signal Process	194	Research	Multiple	BCI	Rehabilitation
Wagner J [23]	2012	Neuroimage	173	Research	Multiple	Robotics	Rehabilitation
Dobkin B [24]	2007	J Physiol	165	Conference	ALS, LiS	BCI	Rehabilitation
Lebedev M and Nicolelis M [25]	2017	Physiol Rev	162	Review	Multiple	BCI	Rehabilitation
Soekadar S et al. [26]	2015	Neurobiol Dis	156	Review	Stroke	BCI	Rehabilitation
Lew E et al. [27]	2012	Front Neuroengineering	153	Research	Stroke	EEG decomposition	Rehabilitation
Elbert T and Rockstroh B [28]	2004	Neuroscientist	152	Review	TBI	Plasticity	Rehabilitation
Obrig H [29]	2014	Neuroimage	151	Review	Multiple	NIRS	Clinical
Ang K et al. [30]	2010	Annu Int Conf IEEE Eng Med Biol Soc EMBC	148	Review	Stroke	BCI	Rehabilitation
Altenmuller E et al. [31]	2009	Ann New York Acad Sci	146	Research	Stroke	Plasticity	Rehabilitation
Ramos-Murguialday A et al. [32]	2012	PLOS One	138	Research	Stroke	BCI	Rehabilitation

(BCI, brain–computer interface; MS, multiple sclerosis; EEG, electroencephalogram; NIRS, near-infrared spectroscopy; SCI, spinal cord injury; TBI, traumatic brain injury; LiS, locked-in syndrome).

**Table 3:** Top-20 scientific production by country.

Country	Articles	Frequency	SCP	MCP	MCP Ratio
USA	96	0.14	76	20	0.21
Italy	91	0.136	73	18	0.19
Germany	66	0.098	38	28	0.42
China	49	0.073	41	8	0.16
United Kingdom	40	0.059	24	16	0.4
Japan	34	0.050	32	2	0.06
Korea	33	0.049	28	5	0.15
Spain	28	0.041	11	17	0.60
Switzerland	22	0.033	14	8	0.36
India	20	0.03	15	5	0.25
Canada	17	0.025	9	8	0.47
Denmark	16	0.023	4	12	0.75
France	15	0.022	7	8	0.53
Austria	13	0.019	9	4	0.31
Poland	13	0.019	12	1	0.078
Australia	11	0.016	6	5	0.45
Brazil	10	0.014	4	6	0.6
Belgium	9	0.013	5	4	0.44
Mexico	9	0.013	9	0	0
Singapore	8	0.011	3	5	0.62

SCP, single country publication; MCP, multiple-country publication.

**Table 4:** Top-20 most-cited authors, sources, and keywords.

Rank	Authors	Citations	Sources	Articles	Keywords	Occurrences
	Name		Name		Words	
1	Pfurtscheller G.	975	IEEE Transactions on Neural Systems and Rehabilitation Engineering	27	neurorehabilitation	147
2	Birbaumer N.	875	Frontiers in Human Neuroscience	23	EEG	115
3	Wolpaw J.R.	501	Frontiers in Neuroscience	22	stroke	105
4	Cohen L.G.	450	Journal of Neural Engineering	20	rehabilitation	78
5	Neuper C.	438	Journal of Neuroengineering and Rehabilitation	19	brain–computer interface	74
6	Mcfarland D.J.	329	Proceedings of the Annual International Conference of the IEEE Engineering in Medicine and Biology Society EMBS	18	motor imagery	64
7	Guan C.	326	Frontiers in Neurology	16	electroencephalography	53
8	Farina D.	286	Neuroscience and Behavioral Physiology	14	BCI	37
9	Hallett M.	284	Neuroimage	11	brain–computer interface	30
10	Ang K.K.	276	Neurorehabilitation and Neural Repair	11	virtual reality	28
11	Blankertz B.	275	Clinical Neurophysiology	10	disorders of consciousness	25
12	Gharabaghi A.	266	IFMBE Proceedings	10	electroencephalography (EEG)	25
13	Scherer R.	259	Neurorehabilitation	10	electroencephalogram	24
14	Makeig S.	237	Restorative Neurology and Neuroscience	10	neurofeedback	23
15	Nitsche M.A.	219	Lecture Notes in Computer Science (Including Subseries Lecture Notes in Artificial Intelligence and Lecture Notes in Bioinformatics)	9	neuroplasticity	23
16	Ramos-Murguialday A.	218	Sensors (Switzerland)	8	transcranial magnetic stimulation	22
17	Paulus W.	215	Annals of Physical and Rehabilitation Medicine	7	brain–computer interface (BCI)	21
18	Pascual Leone A.	205	Frontiers in Systems Neuroscience	7	brain–computer interface	19
19	Schalk G.	191	Neural Plasticity	7	brain–machine interface	18
20	Laureys S.	189	Biomedical Signal Processing and Control	6	minimally conscious state	17

**Table 5:** Cluster analysis based on author’s keyword co-occurrence.

Node	Cluster	Betweenness	Closeness	Page Rank
brain–computer interface (BCI)	1	2.19	0.01	0.01
electroencephalography (EEG)	1	2.74	0.01	0.01
motor imagery (mi)	1	0.00	0.01	0.01
BCI	2	2.98	0.01	0.03
EEG	2	215.55	0.01	0.08
fMRI	2	0.42	0.01	0.01
p300	2	0.00	0.01	0.00
virtual reality	2	5.73	0.01	0.02
brain–computer interface	2	0.84	0.01	0.02
EMG	2	0.00	0.01	0.01
neurorehabilitation	2	0.00	0.01	0.01
cerebral palsy	2	0.13	0.01	0.01
disorders of consciousness	3	11.57	0.01	0.02
traumatic brain injury	3	3.13	0.01	0.01
minimally conscious state	3	4.93	0.01	0.02
vegetative state	3	7.70	0.01	0.02
outcome	3	0.63	0.01	0.01
prognosis	3	0.00	0.01	0.01
coma	3	0.38	0.01	0.01
unresponsive wakefulness syndrome	3	0.00	0.01	0.01
neurorehabilitation	4	513.51	0.02	0.13
brain–machine interface	4	0.49	0.01	0.01
brain–computer interface	4	42.45	0.01	0.04
electroencephalography	4	17.28	0.01	0.06
transcranial magnetic stimulation	4	1.38	0.01	0.01
electroencephalogram (EEG)	4	0.13	0.01	0.01
motor imagery	4	27.94	0.01	0.05
neurofeedback	4	2.39	0.01	0.02
event-related desynchronization	4	0.00	0.01	0.01
motor learning	4	0.16	0.01	0.01
functional near-infrared spectroscopy	4	0.02	0.01	0.01
electroencephalogram	4	0.89	0.01	0.01
spinal cord injury	4	0.24	0.01	0.01
brain-robot interface	4	0.00	0.01	0.01
functional connectivity	4	0.08	0.01	0.01
brain–computer interfaces	4	0.00	0.01	0.01
functional electrical stimulation	4	0.91	0.01	0.01
neuromodulation	4	0.00	0.01	0.01
stroke	5	132.77	0.01	0.09
rehabilitation	5	78.27	0.01	0.05
multiple sclerosis	5	0.00	0.01	0.00
neuroplasticity	5	1.04	0.01	0.01
plasticity	5	0.83	0.01	0.01
motor cortex	5	0.04	0.01	0.01
noninvasive brain stimulation	5	0.43	0.01	0.01
TDCS	5	0.64	0.01	0.01
transcranial direct current stimulation	5	1.60	0.01	0.01
motor control	5	0.00	0.01	0.00
exoskeleton	5	0.47	0.01	0.02
brain–computer interface	5	0.12	0.01	0.01

The cluster analysis was based on centrality measures, including betweenness, closeness, and page rank. Betweenness refers to the number of the shortest paths passing through a given node. The higher the betweenness centrality of the node, the greater the ability to control information passing between the other nodes. The closeness is used to measure the distance of one node to other nodes in a network. Nodes with high closeness centrality obtain information better than do other nodes or tend to have a more direct influence on other nodes.

## Data Availability

Not applicable.

## Abbreviations

ALS, amyotrophic lateral sclerosis; BCI, brain–computer interface; DWT, discrete wavelet transform; ECoG, electrocorticography; EMD, empirical mode decomposition; EEG, electroencephalography; fMRI, functional magnetic resonance imaging; IRB, Institutional Review Board; LiS, locked-in syndrome; MCP, multiple-country production; NIRS, near-infrared spectroscopy; SCI, spinal cord injury; SCP, single country production; SCPs, slow cortical potentials; sTBI, severe traumatic brain injury; WPD, wavelet packet decomposition.
